# Comparative Transcriptome Analysis of Tolerant and Sensitive Genotypes of Common Bean (*Phaseolus vulgaris* L.) in Response to Terminal Drought Stress

**DOI:** 10.3390/plants12010210

**Published:** 2023-01-03

**Authors:** Mayavan Subramani, Carlos A. Urrea, Rasheed Habib, Ketaki Bhide, Jyothi Thimmapuram, Venu Kalavacharla

**Affiliations:** 1 Molecular Genetics and Epigenomics Laboratory, College of Agriculture, Science and Technology (CAST), Delaware State University, Dover, DE 19901, USA; 2 Panhandle Research and Extension Center, University of Nebraska, 4502 Avenue I, Scottsbluff, NE 69361, USA; 3 Bioinformatics Core, Purdue University, West Lafayette, IN 47907, USA

**Keywords:** transcriptomics, common bean, root, leaf, terminal drought stress, tolerant genotypes, sensitive genotypes

## Abstract

We conducted a genome-wide transcriptomic analysis of three drought tolerant and sensitive genotypes of common bean to examine their transcriptional responses to terminal drought stress. We then conducted pairwise comparisons between the root and leaf transcriptomes from the resulting tissue based on combined transcriptomic data from the tolerant and sensitive genotypes. Our transcriptomic data revealed that 491 (6.4%) DEGs (differentially expressed genes) were upregulated in tolerant genotypes, whereas they were downregulated in sensitive genotypes; likewise, 396 (5.1%) DEGs upregulated in sensitive genotypes were downregulated in tolerant genotypes. Several transcription factors, heat shock proteins, and chaperones were identified in the study. Several DEGs in drought DB (data Base) overlapped between genotypes. The GO (gene ontology) terms for biological processes showed upregulation of DEGs in tolerant genotypes for sulfate and drug transmembrane transport when compared to sensitive genotypes. A GO term for cellular components enriched with upregulated DEGs for the apoplast in tolerant genotypes. These results substantiated the temporal pattern of root growth (elongation and initiation of root growth), and ABA-mediated drought response in tolerant genotypes. KEGG (kyoto encyclopedia of genes and genomes) analysis revealed an upregulation of MAPK (mitogen activated protein kinase) signaling pathways and plant hormone signaling pathways in tolerant genotypes. As a result of this study, it will be possible to uncover the molecular mechanisms of drought tolerance in response to terminal drought stress in the field. Further, genome-wide transcriptomic analysis of both tolerant and sensitive genotypes will assist us in identifying potential genes that may contribute to improving drought tolerance in the common bean.

## 1. Introduction

The common bean (*Phaseolus vulgaris* L.) is grown extensively in various regions, from lowland tropical to semi-arid environments, either as a monoculture or intercropped. As a legume, it is an abundant source of total protein, micronutrients, and energy [1]. In the event of terminal or intermittent drought, common bean yield can be negatively affected by up to 60%, a loss that is only further worsened when soil moisture is reduced to 60–70% during grain filling [2,3,4]. Plants employ three key strategies to adapt to drought conditions and respond appropriately. Drought escape through changes in molecular mechanisms that allow them to adapt to the environment, drought avoidance through modifications to morphological and physiological characteristics, and drought tolerance by protein stabilization and osmotic adaptations to cope with dehydration [5]. As a result, a combination of morphological, physiological, biochemical, and molecular changes is more than likely to be induced by changes in the upregulation of several regulatory and functional genes to sense and respond to drought stress [6].

Studying signal perception, gene expression and regulation, and metabolic pathways followed during drought stress is crucial for understanding how plants respond to drought stress [7]. Furthermore, analysis of gene enrichment in metabolic pathways under drought stress will aid in screening for potential genes and drought response mechanisms in diverse plants [8]. It is essential to understand plants’ common and specific gene expression and regulation during drought stress [9]. Due to metabolic and biological processes differing between tissues above and below ground [5], energy production in the leaves is fine-tuned based on the water availability in the roots. Understanding how plants respond to drought stress can be improved by investigating differential gene expression patterns between the tissues above and below ground [10]. Such a study may lead to the discovery of a coordinated biological process, or a distinct pattern of processes followed by each type of tissue. Therefore, a better understanding of drought-specific biological processes, and crosstalk between the regulatory mechanisms of different tissues may contribute significantly towards advancing knowledge of the general molecular mechanisms underlying drought response [11].

Next-generation sequencing, such as RNA sequencing (RNA-Seq), has become increasingly common in analyzing plants’ transcriptome under drought stress. The Andean [12] and Mesoamerican [13] common bean genomes have been sequenced, with a total of 587 megabase pairs (Mbp) and 549.6 Mbp, respectively. These studies provide insight into the genetic basis of biotic and abiotic stress responses [12]. Therefore, genomic information favors the identification of genes that are drought responsive through the use of transcriptomic analysis [2].

Different genotypes of common bean have been studied for differences in gene expression, co-expression of genes, and the relationship between several pathways and the biological functions of specific genes during drought stress [2,14,15]. Genome-wide gene expression analysis in the contrasting genotypes supports the identification of genetic interactions responsible for diverse drought response patterns, genotype-specific drought responses, and possible candidate genes for breeding drought tolerance [16,17,18]. Studying drought responses in field conditions is likely to contribute to a better understanding of the molecular mechanisms underlying drought responses and facilitate the development of effective drought mitigation strategies [19].

Tolerant chickpea (*Cicer arietinum*) genotypes were found to be able to conserve water during terminal drought stress, which was later utilized in reproductive stages. Yet there were no significant differences in root traits between tolerant and sensitive genotypes. Interestingly, roots temporal pattern of water uptake relates to drought tolerance [19,20]. Another study showed that the root-to-shoot ratio increased when plants were exposed to the terminal and intermittent drought stress [21]. Thus, root and shoot growth are highly coordinated in water deficit conditions [22]. In a comparison of differential gene expression in response to the onset of water stress between three hybrid *Brachiaria* genotypes, it has been suggested that faster root growth can offer advantages to the plant in terms of extracting water from soil during a short period of rain [23].

In leaves of the signal grass (*Brachiaria* (Trin.) Griseb.) genotypes, a GO term for carbohydrate and cell wall metabolism was enriched by upregulated DEGs compared to downregulated DEGs in tolerant genotypes. Alternatively, DEGs for apoplastic peroxidase activities, which involved lignification and elasticity of secondary cell walls, were significantly downregulated in the roots of all genotypes, suggesting the pattern of gene expression and the root structure may be influential in response to water stress [10]. In the present study, it is anticipated that root DEGs derived from tolerant genotypes might be enriched with respect to the temporal pattern of root activities, which include variation in cellular response and signaling pathways when responding to drought stress, specifically terminal drought stress in field conditions.

Our study provides genome-wide transcriptomic analysis of six genotypes, three tolerant and three sensitive, grown under terminal drought stress to drought conditions. Our previous study tested these genotypes at two locations for broad temperate and tropical adaptations [24]. Terminal drought stress (after flowering) was imposed on all genotypes in the field. The transcriptomes of both leaves and roots have been generated, and comparisons have been made between tolerant roots and leaves and sensitive roots and leaves. It is anticipated that the described approach will lead to greater insight into the molecular mechanisms of drought tolerance in common bean genotypes.

## 2. Materials and Methods

### 2.1. Field Experiment

**Figure 1:**
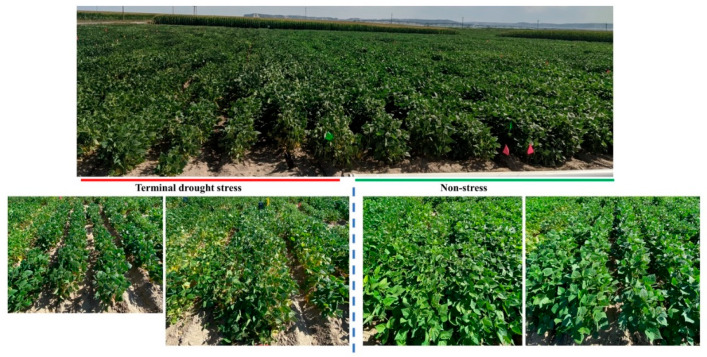
Experiments conducted under stress and non-stress conditions in the field.

Six genotypes of common bean (*Phaseolus vulgaris* L.) were used in the present study. Tolerant genotypes: Merlot’//05F-5055-1/98020-3-1-6-2 (SB-DT3), and USPT-ANT//‘Matterhorn’/98078-5-15-1(SB-DT2) [24], Matterhorn [25], and three sensitive genotypes: Sawtooth [26], Merlot [27], and Stampede [28]. The field experiment was conducted as described previously [29] (Figure 1). A field of silt loam soil (Typic Ustorthents) was used for the cultivation of the common bean genotypes (41°56.6′ N, 103°41.9′ W, 1240 m elevation). There was 75% silt, 15% sand, and 10% clay in the soil. In addition, it had a cation exchange capacity of 17 meq/100 g, a pH of 7.8, and an organic matter content of 14 mg/g. Since the field contained 20.5 kg of residual nitrogen and the manure credit contained 25.9 kg of nitrogen, no additional nitrogen was applied to the field. Most plants reached anthesis (flowering) before the drought was imposed. Until then, the plants were irrigated by drip irrigation. The precipitation totaled 135.1 mm before flowering. This includes irrigation of the plants twice (101.6 mm) and precipitation of 33.5 mm. The rainfall after flowering totaled 16.3 mm, and the plants were not irrigated. Upon reaching almost physiological maturity, the leaves and roots of the plants were collected and frozen in liquid nitrogen. The samples were collected from three sensitive and three tolerant genotypes grown under terminal drought stress in three replicates, resulting in 18 samples [(3 × 3) + (3 × 3)]. Agronomic characters of the tolerant and sensitive genotypes as well as their starch and fat contents were analyzed in our previous study [29], Subramani et al., 2022. For each genotype, RNA isolation and library preparation were performed separately.

### 2.2. RNA Isolation and cDNA Synthesis

Total RNA was isolated from leaves and roots using the Spectrum Total Plant RNA kit (Sigma-Aldrich, St Louis, MO, USA) according to the manufacturer’s instructions. Genomic DNA contamination was eliminated by using DNase I (Invitrogen, Waltham, MA, USA). RNA concentration and purity were determined by the Nanodrop 2000 spectrophotometer (Thermo Scientific, Wilmington, DE, USA). Most RNA samples had a 260/280 ratio between 2 and 2.1. The RNA integrity was then assessed with agarose gel electrophoresis and Agilent Bioanalyzer 2100 (Agilent Technologies, Santa Clara, CA, USA). The complementary DNA (cDNA) was synthesized from 1μg of total RNA according to the manufacturer’s instructions using the ProtoScript II First Strand cDNA Synthesis kit (New England Biolabs, Ipswich, MA, USA).

### 2.3. Library Preparation and Sequencing

The RNA-Seq libraries were prepared using the Illumina TruSeq Stranded mRNA Sample Preparation Kit (Illumina Inc., San Diego, CA, USA). Briefly, Poly(A) tail mRNA was enriched for first-strand c-DNA synthesis. Following the second strand synthesis, multiple washes were carried out for the end repairs. A single A nucleotide was then added to the 3′ ends. PCR was performed to enrich both ends of the shorter fragments with adapters. The cDNA fragment pools were then loaded, and a paired-end read of 150 bp sequencing was performed on the Illumina HiSeq^TM^ 2500 at the Delaware Biotechnology Institute in Newark, DE, USA.

### 2.4. Pre-Processing RNA- Seq Data

*Phaseolus vulgaris* v2.1, downloaded from phytozome v13, was used for genome and gene references. The following URLs were accessed on 8 September 2021. ASTQC (v0.11.9; http://www.bioinformatics.babraham.ac.uk/projects/fastqc/) was used to assess sequence quality, and Trim Galore (v 0.6.5; http://www.bioinformatics.babraham.ac.uk/projects/trim_galore/) was used to trim adapters and reads based on Phred33 score 30, and reads of at least 75 bases were retained. The quality-trimmed reads were mapped to the STAR-indexed genome using STAR (v2.7.9a) [30]. The STAR mapping results, and annotation files (GTF/GFF) were used for reference genome as input for the HTSeq package (version 0.13.5) [31] to calculate the read counts for each gene feature for each sample. For htseq-count, -m union and the -s reverse were used. A read count matrix for all samples was generated by merging counts from all samples with custom Perl scripts. 

### 2.5. Differentially Expressed Genes (DEGs) Identification

DEGs (differentially expressed genes) were identified using DESeq2 (v 1.32.0) [32]. Pairwise comparisons were made between Tolerant root vs leaves and Sensitive root vs leaves. A read count matrix generated using HTSeq was used as input for DESeq2. Genes with a non-zero read count in at least one sample were selected. Then zeros in the matrix were replaced by ones to avoid infinite values being calculated for fold change. DESeq2 uses the negative binomial distribution based on the data model and performs specific estimate variance–mean tests. DESeq2 determines significant DEGs using the Wald test. *p*-value and adjusted *p*-values of false discovery rate (FDR) to correct for multiple tests were added using DESeq2 based on the Wald test.

### 2.6. Functional Analysis of DEGs

ClusterProfiler (Version 4.0.5) [33], and R package (R Version 4.1.0) were used to perform Gene Ontology (GO) and Kyoto Encyclopedia of Genes and Genomes (KEGG) enrichment of significant differentially expressed genes in all pairwise comparisons (padj < 0.05 and log2 FC +/−2). Only the DEGs lists were included for analysis. To perform GO analysis of corresponding DEGs, the Log_2_FC was included as input for agriGO v2 [34] using Singular Enrichment Analysis (SEA) and parametric Analysis of Gene Set Enrichment (PAGE) tools. We selected *Phaseolus vulgaris* species and custom GO database of *P. vulgaris* v 2.1 genome (downloaded from Phytozome v13) (accessed on 8 September 2021) and default parameters such as complete GO database, significance cutoff 0.05, and Hochberg (FDR) method as multiple test correction methods to perform agriGO. The statistically significant GO terms associated with DEGs were derived. 

### 2.7. Volcano Plots

Volcano plots were generated to illustrate a pattern of significant DE genes using the “EnhancedVolcano” R package (v1.10.0) (R version 4.1.0) (accessed on 16 November 2021) using default options/parameters. The pattern and number of significant DE genes were selected based on log2FC and padj < 0.05.

### 2.8. Quantitative Real-Time (qRT-PCR)

QRT-PCR was performed to validate the RNA-Seq results. The C-DNA was synthesized from the root RNA samples. The primers were designed for the up and downregulated DEGs. The list of primers and the corresponding genes were provided in Supplementary Table S6. The primers were designed using the primerQuest™ Tool (Integrated DNA Technologies (IDT), Coralville, IA, USA). The primers were validated by conventional PCR before being used in qRT-PCR. The qRT-PCR was performed on the ABI 7500 real-time PCR (Applied Biosystems, Foster City, CA, USA). Each reaction was carried out in a 25 μL master mix containing 100 ng of C-DNA, 10 μM each of forward and reverse primers, and 12.5 μL of SYBR Green PCR Master Mix (Germantown, MD, USA). The reaction was run at an initial denaturation of 95 °C for 10 min, followed by 40 cycles at 95 °C for 15 s and a final extension at 60 °C for 1 min. The samples were run in triplicate, and the actin gene [35] was used to normalize qPCR samples and as an internal control. The comparative CT method 2^−ΔCT^ [36] was used to calculate relative expressions.

## 3. Results

### 3.1. Data Quality and Summary of Reads

A total of 4615 million reads were obtained from the tolerant and sensitive root and leaf samples. There were approximately 129 million reads from each sample (Table 1). More than 92% of reads were mapped to the reference genome (*Phaseolus vulgaris* v2.1). These confirmed the high data quality and could be used for further analysis. In order to visualize the variation in RNA sequences between root and leaf samples, principal component analysis was performed with DESeq2. There was a clear distinction between root and leaf replicates from tolerant and sensitive genotypes on the PCA scatter plot (Supplementary Figure S1). In addition, this will provide further confirmation that there is great variation in gene expression between the roots and leaves.

### 3.2. Specific Gene Expression within Tolerant and Sensitive Genotypes

**Figure 2:**
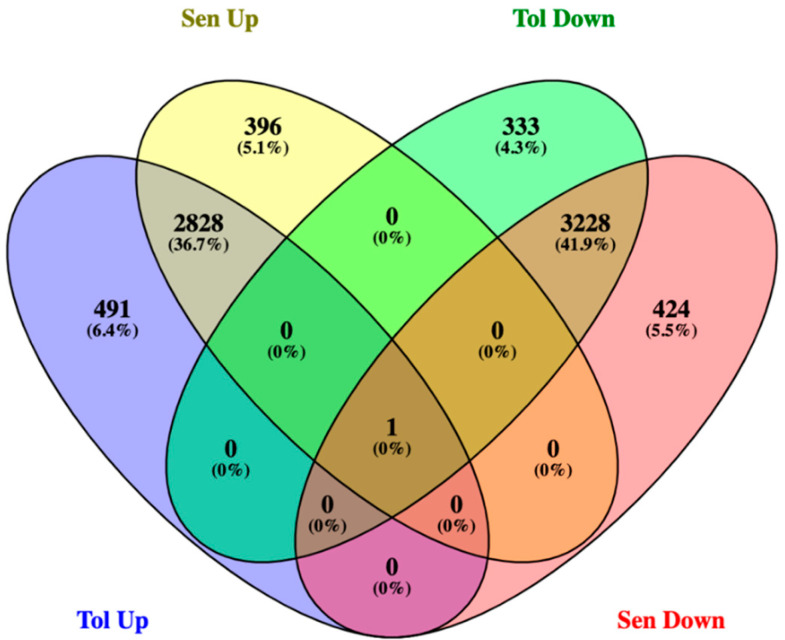
Venn diagram shows the potential upregulated and downregulated DEGs in tolerant and sensitive genotypes. DEGs were quantified based on log2 fold changes ±2 and FDR < 0.05.

**Figure 3:**
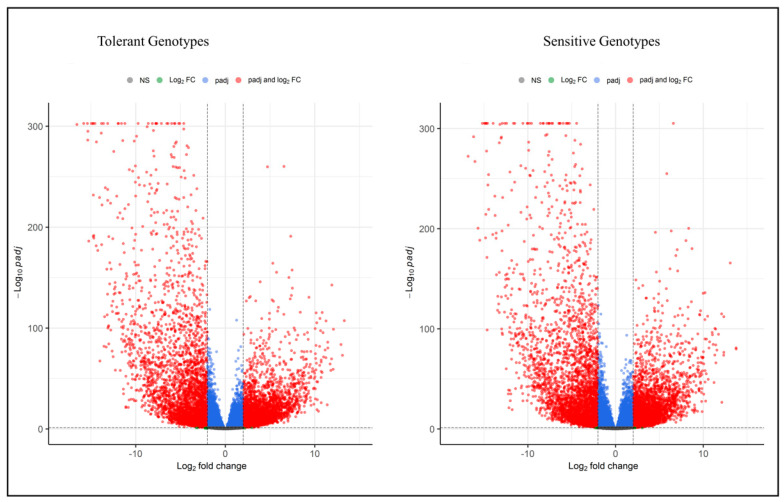
Volcano plots illustrate the up and downregulation of DEGs in tolerant and sensitive genotypes. The red color indicates the up and down regulation of DEGs based on the log2 fold change and padj < 0.05.

Using the two pairwise comparisons (Tolerant genotypes roots vs. leaves, Sensitive genotypes roots vs. leaves), the total number of responsive genes identified with the restriction to FDR < 0.05 was 15,685 for the tolerant and 16,226 for the sensitive genotypes (Supplementary Tables S1 and S2). With FDR < 0.05 and log 2FC ± 2, 491 (6.4%) upregulated DEGs were unique to the tolerant genotype. Similarly, 396 (5.1%) were enriched in sensitive genotypes (Figure 2). These DEGs are likely to play an important role in response to terminal drought. Furthermore, the scatter plot was constructed based on the log2fold change and −log_10_ padj to determine the upregulated and downregulated genes by restricting FDR < 0.05 and log2fold change ±2. In total, 3319 DEGs were upregulated, and 3561 genes were downregulated in the tolerant genotypes comparisons. In the sensitive genotypes comparisons, 3224 DEGs were upregulated, and 3652 were downregulated (Figure 3).

### 3.3. GO Enrichment Analysis

**Figure 4:**
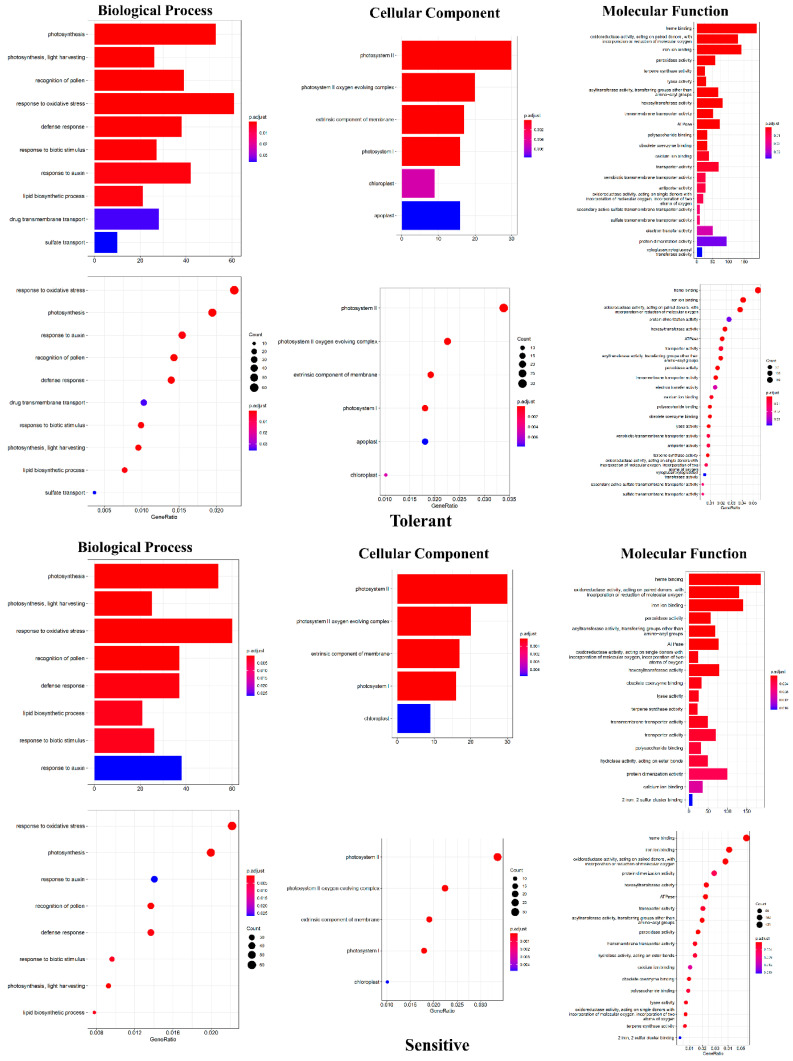
Based on gene ontology (GO) annotation, the root and leaf comparisons of tolerant and sensitive genotypes reveal DEG enrichment for biological processes, cellular components, and molecular functions. Gene ratios for enriched DEGs are included. The number of genes involved are plotted against each GO terms (Biological process, cellular components, molecular function). The DEGs were annotated against the GO database. The GO enrichment was based on the padj < 0.05.

A GO annotation analysis was performed to analyze DEGs based on padj < 0.05, associated with cellular components, molecular functions, and biological processes. In both the genotypes comparisons, biological process enrichment revealed that DEGs enriched in photosynthesis, light harvesting, response to oxidative stress, recognition of pollen, defense response, lipid biosynthetic process, response to biotic stimulus, and response to auxin (Figure 4). Among the selected DEGs 2726 of the tolerant genotypes comparisons, 345 genes were involved in diverse biological processes. In contrast, out of 2706 DEGs, 303 genes were involved in biological processes for the sensitive genotypes. It was found that sulfate transport and drug transmembrane transport were the two biological processes enriched specifically in tolerant genotype comparisons (Figure 4). The gene ratios were almost identical between the two genotypes for each biological process (Supplementary Table S3). Among 3576 DEGs, 133 and 92 were enriched for cellular component function for tolerant and sensitive genotype comparisons, respectively. The DEGs specific to cellular components, such as the apoplast, were enriched in the tolerant genotypes compared to the sensitive genotypes. In each cellular component function, the gene ratios were similar. However, the tolerant genotypes were more enriched than the sensitive genotypes for the molecular function category. These include xenobiotic transmembrane transporter activity, antiporter activity, secondary active sulfate transmembrane transporter activity, sulfate transmembrane transporter activity, electron transfer activity, and xyloglucan:xyloglucosyl transferase activity. Also, specific molecular functions enriched for sensitive genotypes were hydrolase activity, acting on ester bonds, and two iron, two sulfur cluster binding. Higher DEGs ratios for heme binding were 189 out of 3448 and 186 out of 3424 for the tolerant and sensitive genotypes, respectively. The differences in the DEGs enrichment may indicate the possible biological functional differences in the tolerant and sensitive genotypes in response to drought stress.

### 3.4. KEGG and DEGs

**Figure 5:**
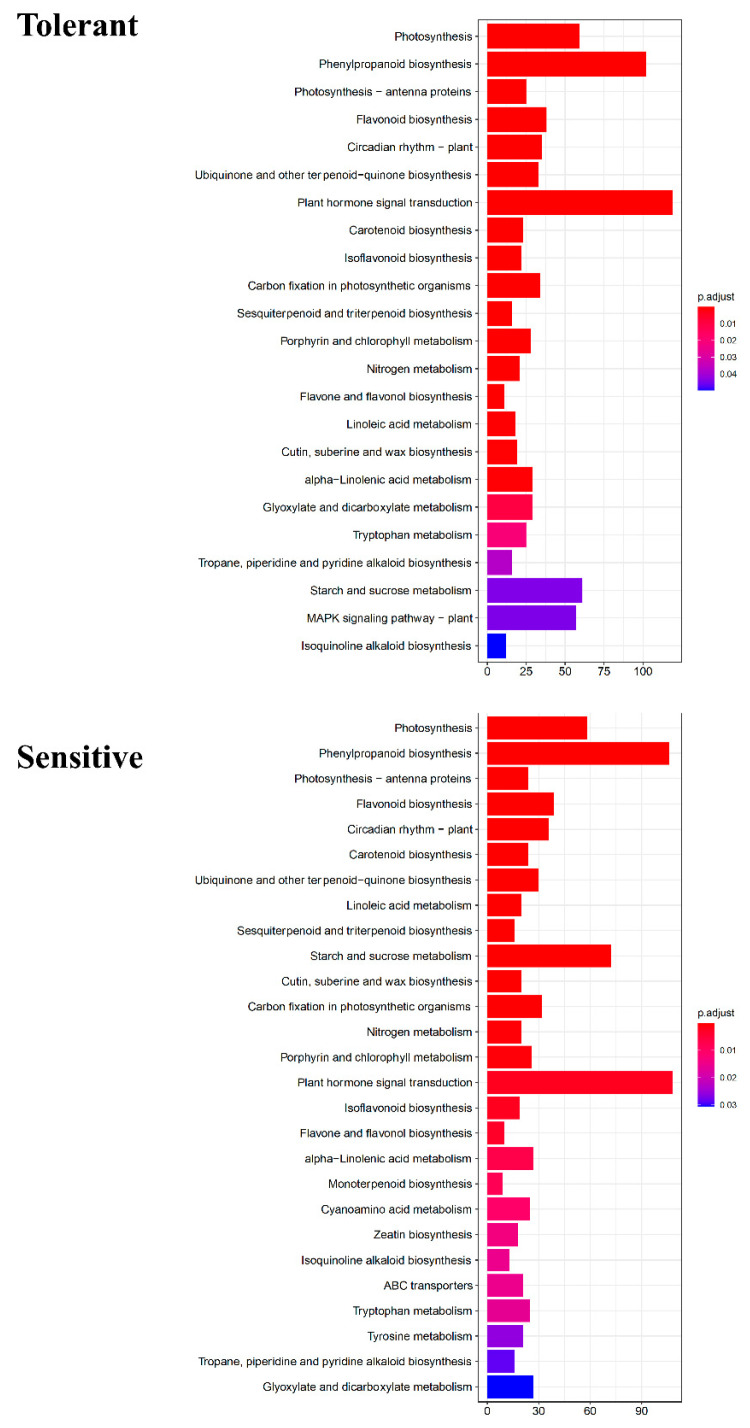
DEG enrichment of the metabolic pathway. There was a difference between the tolerant and sensitive genotypes in the number of genes enriched for the particular pathways.

In addition, KEGG enrichment function analyses were performed for both the tolerant and sensitive genotype comparisons. In both the tolerant and sensitive genotype comparisons, the number of genes enriched for the plant hormone signal transduction pathway followed by phenylpropanoid biosynthesis was higher than the other pathways (Figure 5). MAPK signaling pathway was specific to tolerant genotype comparisons, while monoterpenoid biosynthesis, cyanoamino acid metabolism, zeatin biosynthesis, ABC transporters, and tyrosine metabolism were exclusive to sensitive genotype comparisons. More pathways in sensitive than tolerant genotype comparisons were identified. DEGs for starch and sucrose metabolism were comparatively upregulated in sensitive genotypes. Differences in the pathways may be associated with specific functional roles in response to drought stress.

### 3.5. DEGs in Drought DB (Data Base)

**Figure 6:**
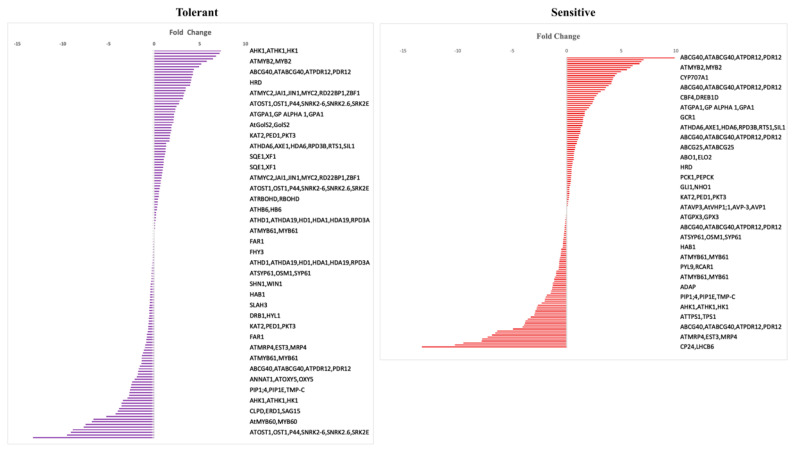
DEGs identified in the tolerant and sensitive genotype comparisons have the best hits to Arabidopsis genes in drought DB. DEGs are shown with fold changes.

It was found that 155 and 154 DEGs from tolerant and sensitive genotype comparisons, respectively, had best hits to *Arabidopsis* genes in the drought database. These lists of genes, their names, and descriptions, identified from phytozome, along with the corresponding log2FC, are included in an Excel file (Supplementary Table S4). Among the DEGs, Phvul.002G107100 and Phvul.001G166500 were the two genes with higher expression (Log2FC > 7) in the tolerant and sensitive genotypes comparisons (Figure 6). The Phvul.002G107100 gene is homologous to AHK1 in *Arabidopsis*. The AHK1 (Histidine kinase 1) gene was reported to regulate drought and salt responses through ABA (abscisic acid) independent and dependent signaling pathways [37]. Similarly, the sensitive genotypes contain the homologous gene Phvul.001G166500 to *Arabidopsis* ABCG40. The *atabcg40* mutants exhibited reduced drought tolerance due to impaired lateral root formation and loss of stomatal function [38]. Upregulated DEGs might play an important role in drought responses associated with ABA in both genotypes’ comparisons.

### 3.6. Transcription Factors (TFs)

Out of 25,419 genes, 1418 TFs were identified in the tolerant genotype comparisons. Similarly, out of 25,466 genes in the sensitive genotypes, 1416 TFs were identified (Supplementary Table S5). The C2H2 family protein of TFs was associated with the upregulated DEGs Phvul.002G075900 and Phvul.002G075900 with Log2FC > 10 in tolerant and sensitive genotypes, respectively. Transcription factors zinc finger protein 7 and LOB domain-containing protein 27 were identified specifically in the tolerant genotypes’ comparisons. In the sensitive genotypes, myb domain protein 82, myb domain protein 21, WOX family protein, homeodomain GLABROUS 12 AGAMOUS-like 58, myb domain protein 101, myb domain protein 64 and zinc finger protein 3 were identified specifically.

### 3.7. Validation of Differentially Expressed Genes

To confirm the results of RNA-Seq, we analyzed the expression patterns of randomly selected up and downregulated differentially expressed transcripts using qRT-PCR. QRT-PCR results for selected DEGs were similar to those obtained by RNA-Seq. Comparisons of the expression patterns revealed a strong correlation (r^2^ = 0.8693) (Supplementary Figure S2) between both techniques.

## 4. Discussion

The incidence of terminal drought stress in common bean is most common in bean growing areas. Drought stress adversely impacts the quality and yield of common bean, particularly during the pod development and seed filling stages [4,39]. Therefore, improving the plant’s ability to withstand this stress is considered a key objective of breeding programs [4,40]. The complete genome sequence of the common bean is now available. This makes it possible to discover transcription regulation patterns and enrich gene databases by exploring transcriptome maps and related pathways among tolerant and sensitive genotypes. In addition to improving drought tolerance, this may also help identify the molecular mechanisms of abiotic stress [2]. Several studies have examined the transcriptional response of common bean to drought stress under controlled conditions [2,15,41,42,43,44]. However, studies that developed and utilized transcriptome data associated with terminal drought stress imposed on field grown common bean genotypes are limited. The primary objective of this study is to identify transcriptional changes and molecular mechanisms underlying the terminal drought stress induced on sensitive and tolerant genotypes of field-grown common beans.

### 4.1. Potential DEGs in Tolerant and Sensitive Genotypes

In total, 486 DEGs that were upregulated in tolerant genotypes were found to be downregulated in sensitive genotypes. It is likely that these DEGs contribute to drought tolerance. Among these genes, Phvul.010G050500, an ethylene-responsive element binding factor 13, has been reported as a drought candidate gene in solanaceous plants and is involved in gene regulation of metabolic pathways [45]. Phvul.010G088500 (S-locus lectin protein kinase family protein) is also known to be expressed highly in rice (*Oryza sativa*) and *Arabidopsis* as a response to stress conditions such as drought, salt, and other biotic stresses [46]. The log2FC of these genes was >5 in tolerant genotypes. Also, the C2H2 and C2HC zinc fingers superfamily protein (Phvul.002G256900) and the ethylene response factor 1 (Phvul.001G160200) both display log2FC > 4 and are upregulated specifically in tolerant genotypes. These transcription factors regulate gene expression in abiotic stresses such as drought and salt genes in *Arabidopsis* [47,48]. Similarly, 392 DEGs upregulated in sensitive genotypes were downregulated in tolerant genotypes. Among the DEGs, Phvul.011G163300, annotated as Ankyrin repeat family protein, had a log2FC > 2. Ankyrin repeat protein (DRA1) is reported to negatively regulate drought tolerance in *Arabidopsis* [49]. The volcano plot analysis indicated that more genes were up and downregulated in the tolerant genotypes than in the sensitive genotypes. In the tolerant genotypes, Phvul.011G182500 had a log2FC > 13 and padj < 0.05. This gene is associated with an MLP-like protein 43 that is highly expressed in roots and cotyledons. It is a positive regulator of drought stress response and regulates ABA-responsive gene expression [50]. Additionally, Phvul.003G285900 is upregulated (Log2Fc > 7) with best hits in drought DB in drought-tolerant genotypes, while its homologous (PCK1) has been reported to influence drought tolerance in *Arabidopsis* by reducing stomatal conductance [51]. Similarly, in sensitive genotypes, Phvul.001G166500 showed upregulation with log2FC > 9, and its homologous PDR12 (pleiotropic drug resistance 12) was reported to improve drought tolerance mediated through efficient ABA transport in *Arabidopsis* [38]. DEGs such as Phvul.008G147800 and Phvul.L002632 were upregulated in both tolerant and sensitive genotypes with log2FC > 13. They belong to the RmlC-like cupins superfamily protein, which is known to function in the biological process of small molecules (metabolites) involved in oxidoreductase and disomerase activities. It has been reported that these metabolites, such as amines and sulfur amino acids, contribute to maintaining osmotic potential and preventing water loss in plants during stress conditions [52]. In addition, a similar gene was identified (Phvul.002G107100) that has the highest hits in drought DB as well as a higher expression level, i.e., log2FC > 7, in both the tolerant and sensitive genotypes. The homologous version of this gene (HK1) has enhanced drought tolerance and indicates ABA-dependent drought response in *Arabidopsis* [37,53].

### 4.2. GO Enrichment

GO enrichment was carried out for the selected DEGs to identify the preferred GO terms based on padj < 0.05. The analysis revealed enrichment in DEGs in response to various stresses or inducers, such as photosystem, oxidative stress, signaling, hormone, biotic stimulus, metabolite transport, defense response, and enzymes. In common bean, these GO terms are primarily enriched during drought stress [2], indicating that DEG enrichment is drought-specific and responsive to terminal drought stress. Among GO terms for biological processes, the DEGs upregulated in the tolerant genotypes specifically are related to sulfate transport and drug transmembrane transport compared to sensitive genotypes. Similarly, Pereira et al., 2020 [2] reported DEG enrichment associated with the formation of sulfur-containing compounds in drought-tolerant genotypes of common bean. Sulfate transport is an important component of abiotic stress responses, such as drought. When plant roots are exposed to abiotic stresses, such as drought, sulfate transport plays a critical role in producing sufficient ABA and glutathione compounds [54]. As part of the ABA biosynthesis process, cysteine is utilized as a sulfur donor via sulfate transport. Additionally, sulfate accumulation in the root system increases cysteine and glutathione compounds, which contribute to root growth during drought stress [55]. Therefore, it is possible that upregulation of sulfate transport in tolerant genotypes is critical for drought tolerance through an increase in ABA synthesis, glutathione, and root growth during terminal drought stress. In addition, DEGs’ enrichment for drug transmembrane transport is elevated in drug-tolerant genotypes. The upregulation of DEGs for drug transmembrane transport has been reported in other plant species such as *Phormium tenax* (New Zealand Flax), *Pinus massoniana* (Masson Pine), and *Boea hygrometrica* [56,57,58] under drought stress as well. In contrast, both genotypes exhibited higher enrichment of gene ratios related to the GO term “response to oxidative stress”.

In the GO term for cellular components, DEG enrichment for apoplast was upregulated in the tolerant genotypes, but downregulated in the sensitive genotypes. In drought-stressed plants, the alkaline nature of apoplast aids in the accumulation of anionic form of ABA to initiate stomata closure [59]. During drought stress, apoplasts are also reported to participate in nutrient transfer from roots and ROS (reactive oxygen species)-mediated cell signaling [60,61]. Moreover, apoplastic ROS can enhance root elongation via loosening cell walls and protect the root from ROS-induced damage during water stress [62]. Thus, apoplasts play a role in endogenous signaling as well as in the initiation of root growth in tolerant genotypes during terminal drought stress. For the molecular function category, DEGs’ enrichment was found to be upregulated for more GO terms such as xenobiotic transmembrane transporter activity, antiporter activity, secondary active sulfate transmembrane transporter activity, sulfate transmembrane transporter activity, electron transfer activity, and xyloglucan:xyloglucosyl transferase activity in tolerant genotypes than in sensitive genotypes. Their role in enhancing drought tolerance has been reported [63,64,65].

### 4.3. KEGG

KEGG pathway enrichment was conducted for significant DEGs in the tolerant and sensitive genotype comparisons. In both genotypes, the number of DEGs associated with plant hormone signal transduction pathways is high, followed by phenylpropanoid biosynthesis. Plant hormones play a key role in regulating plant growth, development, and response to biotic and abiotic stresses. Several plant hormones, including ABA, jasmonic acids (JA), ethylene (ET), and salicylic acids (SA), may act as mediators in the prevention or response to drought stress [66]. The hormones work through a coordinated network of signal transduction and cross-talk between hormones to facilitate the switchover of pathways to cope with drought stress [67]. Similarly, Wang et al. (2020) [68] report that the shared DEGs in response to drought stress indicate overlap and crosstalk between hormone signal transduction pathways in Tobacco (*Nicotiana tabacum*).

Phenylpropanoids, such as flavonoids, isoflavonoids, plant hormones, anthocyanins, and lignins, play an important role in biotic and abiotic stress [69]. DEGs associated with the phenylpropanoid pathway were enriched in tolerant genotypes in this study. A similar enrichment of DEGs for the phenylpropanoids pathway has been reported in foxtail millet (*Setaria italica* (L.) P. Beauv.) under drought stress [70].

However, DEGs’ enrichment for the MAPK signaling pathway was upregulated in tolerant genotypes. Mitogen-activated protein kinases (MAPKs) are signal transduction modules that transmit extracellular signals into cells. They regulate gene expression by phosphorylating many transcription factors. A number of MAPK components, including MAPK kinase and MAPK kinase kinase, contribute to the development of cells, hormonal activities, and biotic and abiotic stresses [71,72]. A drought-induced MAPK pathway enrichment with upregulated DEGs has been reported for pearl millet (*Pennisetum glaucum* (L.) [73]. There is also a possibility that in this study, the interaction of enriched pathways, such as the plant hormone signaling pathway and MAPK pathways, can positively impact drought tolerance in tolerant genotypes, as they share common byproducts [74].

DEGs enrichment for monoterpenoid biosynthesis was upregulated in sensitive genotypes. Plant terpenes are synthesized in the cytosol as well as plastid through the mevalonate (MVA) and 2C-methyl-D-erythritol-4-phosphate pathway (MEP) [75]. Terpenoid metabolites have been reported to protect plants against biotic and abiotic stresses [76]. Field drought stress conditions were found to modulate monoterpenoid biosynthesis. Long-term and severe field drought stress resulted in the downregulation of DEGs that encode structural enzymes for monoterpenoid biosynthesis [77]. This may explain the downregulation of DEGs for monoterpenoid biosynthesis in tolerant genotypes in this study. Similarly, Wan et al. (2022) [78] reported that DEGs downregulated for monoterpenoid biosynthesis in alfalfa (*Medicago sativa* L.) under drought stress.

## 5. Conclusions

We analyzed comparative transcriptomes of roots and leaves of tolerant as well as sensitive genotypes of field-grown common beans under terminal drought stress. DEG regulation was significantly different between tolerant and sensitive genotypes, with downregulated DEGs significantly higher in sensitive genotypes than upregulated DEGs. DEGs overlapping between genotypes were also found in the drought database. Several transcription factors, heat shock proteins, and chaperones were identified in both genotype comparisons. ABA-regulated drought stress responses were associated with DEGs with higher expression in both genotypes. It was found that DEGs in the tolerant genotype were involved in signal transduction, oxidative stress damage, and transportation. The enrichment of DEGs for biological and cellular components’ GO terms in tolerant genotypes may further explain the temporal pattern of root growth and the ABA-dependent drought tolerance. KEGG pathway analysis indicates that drought-tolerant genotypes exhibit crosstalk between pathways. It is likely that transcription factors associated with pathways, including ERF, BHLH, EIL, and bZIP, contribute to drought tolerance in response to terminal drought stress. The pairwise transcriptomic approaches revealed molecular signatures such as upregulated DEGs enriched with cellular components and biological processes for apoplasts, sulfate, and drug membrane transport in tolerant genotypes, compared to sensitive genotypes. KEGG analysis revealed MAPK and plant hormone signaling pathways interaction in tolerant genotypes. These molecular signatures may contribute to the development of genotypes’ tolerance against terminal drought stress. In the future, this study may also serve as a reference for understanding the drought stress transcriptomes of other legume species.

## Figures and Tables

**Table 1:** Summary of reads with average read counts for the individual samples and the cumulative totals of both roots and leaves.

Tolerant Root		Tolerant Leaves		Sensitive Root		Sensitive Leaves	
**Matt_7R**	139,640,906	Matt_10L	107,711,956	Swa_4R	130,624,430	Swa_2L	130,454,386
Matt_7R	138,965,052	Matt_14L	116,817,638	Swa_5R	133,482,394	Swa_6L	110,347,824
Matt_9R	154,185,446	Matt_17L	117,538,996	Swa_6R	152,247,298	Swa_9L	125,654,646
**Total**	**432,791,404**	**Total**	**342,068,590**	**Total**	**416,354,122**	**Total**	**366,456,856**
**Average**	**144,263,801**	**Average**	**114,022,863**	**Average**	**138,784,707**	**Average**	**122,152,285**
MerX_10R	174,025,346	MerX_11L	162,408,546	Mer_1R	104,058,518	Mer_1L	160,053,034
MerX_11R	119,788,128	MerX_15L	119,504,092	Mer_2R	120,540,136	Mer_5L	144,966,788
MerX_12R	106,557,634	MerX_16L	110,960,258	Mer_3R	112,068,328	Mer_7L	128,432,570
**Total**	**400,371,108**	**Total**	**392,872,896**	**Total**	**336,666,982**	**Total**	**433,452,392**
**Average**	**133,457,036**	**Average**	**130,957,632**	**Average**	**112,222,327**	**Average**	**144,484,131**
USPT_13R	84,249,968	USPT_12L	168,923,136	Stam_16R	115,711,432	Stam_3L	110,748,340
USPT_14R	128,161,580	USPT_13L	107,542,292	Stam_17R	102,825,568	Stam_4L	140,862,648
USPT_15R	121,624,450	USPT_18L	138,913,792	Stam_18R	144,821,904	Stam_8L	129,873,270
**Total**	**334,035,998**	**Total**	**415,379,220**	**Total**	**363,358,904**	**Total**	**381,484,258**
**Average**	**111,345,333**	**Average**	**138,459,740**	**Average**	**121,119,635**	**Average**	**127,161,419**

Matt—Matterhorn; MerX—MerlotX; Swa—Sawtooth; Mer—Merlot; Stam—Stampede.

## Data Availability

Data is contained within the article or supplementary material.

## Supplementary Materials

The following are available online at https://www.mdpi.com/article/10.3390/plants12010210/s1, Supplementary Figure S1: Principle component analysis indicates differences between the replicates of leaves and roots, as well as root samples differing from leaf samples from each genotype; Supplementary Figure S2: (a) Comparison of qRT-PCR and RNA-Seq results for the selected DEGs. Data shown are the mean value of triplicates ±SD (b) Correlation between qRT-PCR and RNA-Seq techniques; Supplementary Table S1 and Table S2: The table shows the up or downregulated DEGs in tolerant and sensitive genotypes compared between roots and leaves with FDR < 0.05; Supplementary Table S3: Comparison of the tolerant and sensitive genotypes in root vs. leaf shows the enrichment of DEGs on biological processes, cellular components, and molecular functions; Supplementary Table S4: A summary of DEG enrichment in the droughtDB is presented in the table along with the identification of the best hit Arabidopsis ID. Most DEGs of tolerant and sensitive genotypes in the drought database overlap; Supplementary Table S5: An analysis of transcription factors and their family of tolerant and sensitive genotypes is presented in the table. DEGs with higher log2FC are similar between the two genotypes; Supplementary Table S6: List of up and downregulated genes and their primer sequences for the validation of RNA-Seq by qRT-PCR.
