# Supplementary material for: Comparative Transcriptome Analysis of Tolerant and Sensitive Genotypes of Common Bean (Phaseolus vulgaris L.) in Response to Terminal Drought Stress
Source: Plants (Basel). 2023 Jan 3;12(1):210. doi: 10.3390/plants12010210 (PMC9824821; doi:10.3390/plants12010210)
Supplement: Supplementary file 1 [file plants-12-00210-s001.zip › figures.pdf]

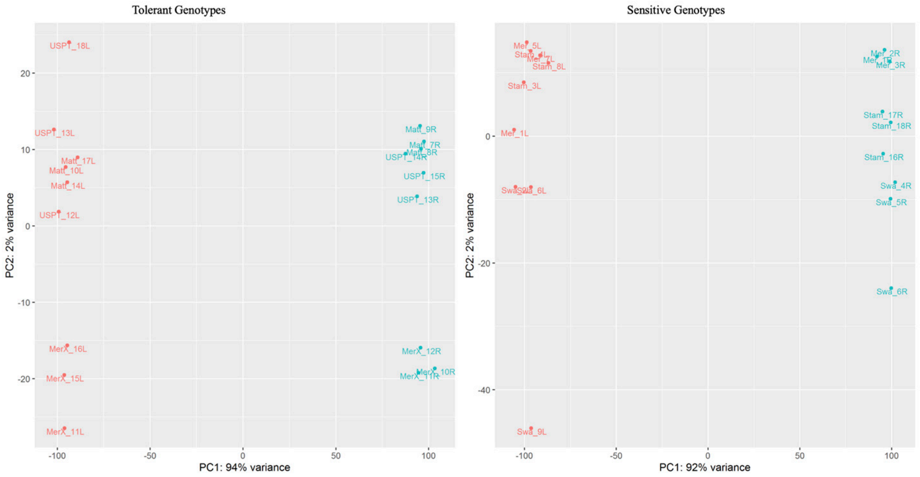

Figure S1: Principle component analysis indicates differences between the replicates of leaves and roots, as well as root samples differing from leaf samples from each genotype.

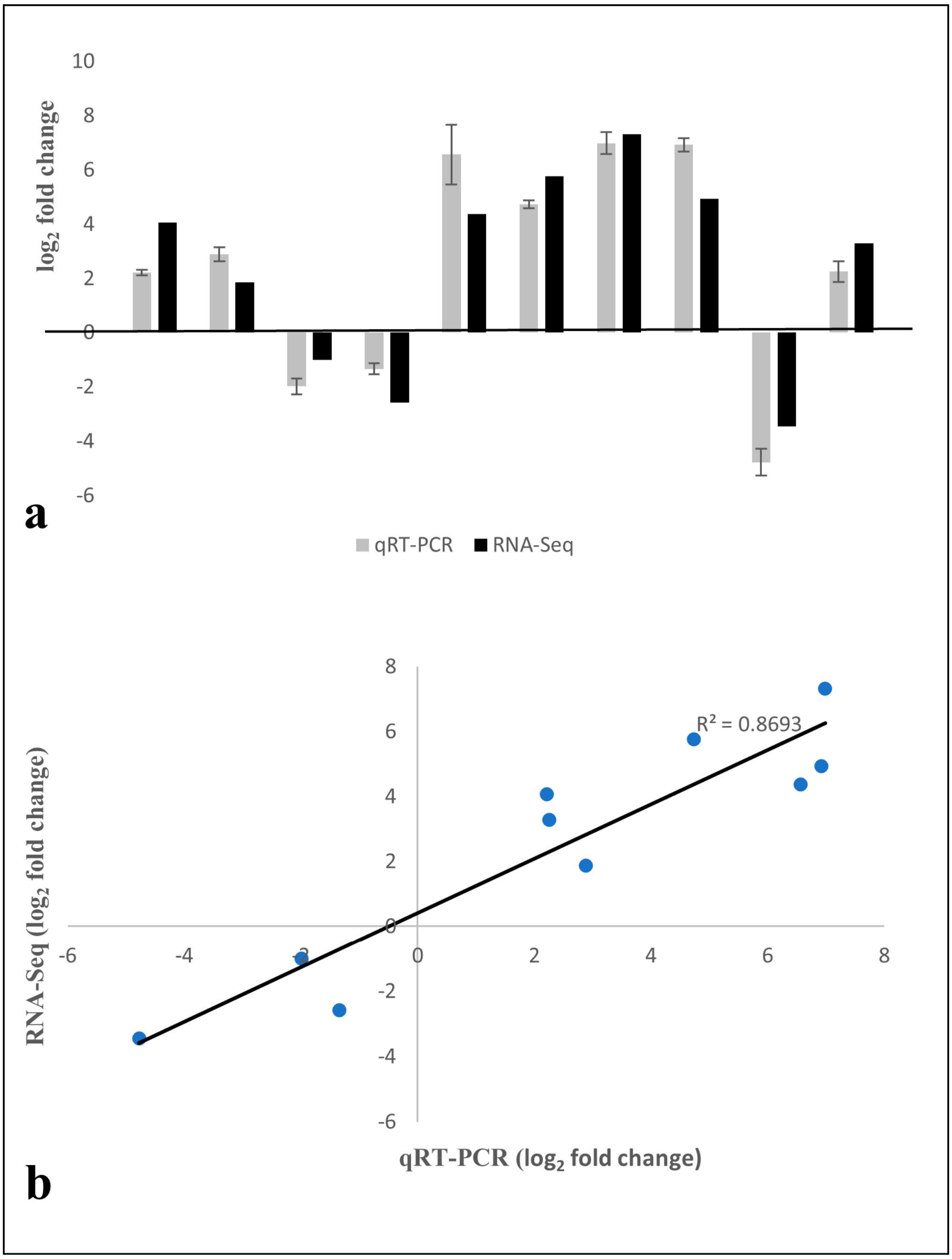

Figure S2: (a) Comparison of qRT-PCR and RNA-Seq results for the selected DEGs. Data shown are the mean value of triplicates ±SD (b) Correlation between qRT-PCR and RNA-Seq techniques.
